# Autophagy and misfolded proteins in neurodegeneration

**DOI:** 10.1016/j.expneurol.2010.11.003

**Published:** 2012-11

**Authors:** Daniel J. Metcalf, Moisés García-Arencibia, Warren E. Hochfeld, David C. Rubinsztein

**Affiliations:** Department of Medical Genetics, Cambridge Institute for Medical Research, Wellcome/MRC Building, Addenbrooke's Hospital, Hills Road, Cambridge CB2 2XY, UK

**Keywords:** Autophagy, Neurodegeneration, Huntington's disease

## Abstract

The accumulation of misfolded proteins in insoluble aggregates within the neuronal cytoplasm is one of the common pathological hallmarks of most adult-onset human neurodegenerative diseases. The clearance of these misfolded proteins may represent a promising therapeutic strategy in these diseases. The two main routes for intracellular protein degradation are the ubiquitin–proteasome and the autophagy–lysosome pathways. In this review, we will focus on the autophagic pathway, by providing some examples of how impairment at different steps in this degradation pathway is related to different neurodegenerative diseases. We will also consider that upregulating autophagy may be useful in the treatment of some of these diseases. Finally, we discuss how antioxidants, which have been considered to be beneficial in neurodegenerative diseases, can block autophagy, thus potentially compromising their therapeutic potential.

## Research highlights

►Autophagy compromise occurs in different neurodegenerative diseases. ►Upregulating autophagy may be useful in the treatment of some neurodegenerative diseases. ►Many different reactive oxygen species scavengers impair autophagy

## Introduction to autophagy

(Macro)autophagy involves the formation of double membrane-bound structures called autophagosomes around portions of cytoplasm and associated organelles. These autophagosomes ultimately fuse with lysosomes, where their contents are degraded (Yang and Klionsky, 2010). Autophagy acts as a starvation response to maintain cellular nutrient levels and helps to regulate intracellular organelle homeostasis. Autophagy also plays an essential role in the removal of toxic/aggregate proteins and damaged organelles, like mitochondria, that would otherwise damage cells during stress.

**Fig. 1:**
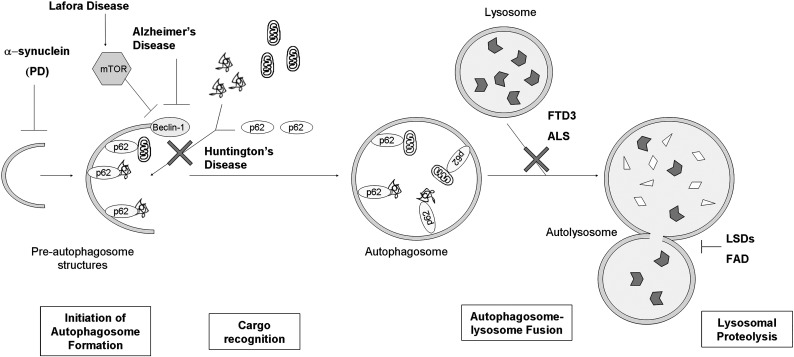
Impairment of different steps of the autophagic pathway in various neurodegenerative diseases. PD, Parkinson's disease; FTD3, frontotemporal dementia linked to chromosome 3; ALS, amyotrophic lateral sclerosis; LSDs, lysosomal storage disorders; FAD, early-onset familial Alzheimer's disease.

Mammalian autophagy can be considered in initiation, elongation, maturation and fusion steps (Fig. 1). Initiation involves the formation of a membrane structure termed the phagophore in the cytoplasm. This is followed by the elongation phase, where the phagophore accumulates additional membrane and expands to enclose a region of cytoplasm, forming an autophagosome. The origins of the membranes during initiation and elongation are still not fully understood, and there may be multiple sources (Hamasaki and Yoshimori, 2010). Membrane could be generated de novo or from existing compartments such as ER (Hayashi-Nishino et al., 2009; Yla-Anttila et al., 2009), mitochondria (Hailey et al., 2010), and the plasma membrane (Ravikumar et al., 2010). Autophagosomes then undergo maturation, which involves recruitment of proteins, microtubule-dependent transport towards the perinuclear region of the cell, and fusion with endosomes to form amphisomes. Finally, autophagosomes/amphisomes and their contents fuse with lysosomes to form autophagolysosomes (also called autolysosomes) (Jahreiss et al., 2008; Mizushima, 2007; Orsi et al., 2010; Ravikumar et al., 2009).

## Autophagy signalling

Autophagosome formation is regulated by many signals that fall into two broad categories: mammalian target of rapamycin (mTOR)-dependent and mTOR-independent.

### mTOR-dependent signalling

The mTOR complex integrates a number of signals that monitor the energy status of cells and negatively regulates autophagosome biogenesis. mTOR can form two types of functional complex. mTOR complex (mTORC) 1 is involved in autophagy, but also regulates other processes like translation and ribosome biogenesis (Jung et al., 2010; Noda and Ohsumi, 1998; Sarbassov et al., 2005).

Cells can monitor and respond to impaired nutrients or energy via a number of mechanisms, including essential amino acid detection via Rag (Sancak et al., 2010, 2008) and activation of AMP-activated protein kinase (AMPK) in response to elevated AMP to ATP ratios (Meijer and Codogno, 2006). Both of these pathways decrease mTOR activity in response to depleted nutrients. Insulin and growth factor signals also feed in to the mTOR pathway via insulin receptors, which ultimately signal to activate mTORC1 (Klionsky and Emr, 2000).

mTORC1 controls autophagy by direct interaction with the Ulk1-Atg13-FIP200 complex. Inhibition of mTOR activates Atg13 kinase activity, leading to phosphorylation of ULK1/2 and FIP200, which in turn induces autophagy (Chan et al., 2009; Hosokawa et al., 2009; Jung et al., 2009).

### mTOR-independent signalling

There are a number of mTOR-independent signals that signal to the autophagy pathway. Reduction of free inositol and myoinositol-1, 4, 5-trisphosphate (IP3) levels results in an upregulation of autophagy (Sarkar et al., 2005). The inositol pathway has been shown to be regulated by cyclic AMP (cAMP), where increasing adenylyl cyclase activity results in more intracellular cAMP. This activates phosopholipase Cε (PLCε) through Epac and Rap2B. PLCε produces IP3, which, in turn, releases calcium from ER stores. Intracytosolic calcium activates calpains which activate Gsα and that results in increased adenylyl cyclase activity and increasing cAMP, completing the potential cycle. How exactly intracytosolic calcium, cAMP and IP3 control autophagy is unclear (Williams et al., 2008).

### PI3P

Beclin-1 is a key regulator of autophagy pathways through its ability to form a number of different complexes. When bound to Bcl2 family member proteins, Beclin-1 is unable to interact with other components of autophagy stimulatory complexes. The Bcl-2 interaction is regulated by nutrient status. In response to starvation, increased JNK1 phosphorylation of Bcl2 disrupts Beclin-1-Bcl2 binding (Wei et al., 2008). In contrast, when incorporated into the class III PI3-K complex, which comprises Vps34, Vps15 and Atg14, Beclin-1 is stimulatory and targets to pre-autophagosome structures. Moreover, Beclin-1 can also become part of a complex that replaces Atg14 with Vps38 and this targets the complex to endosomes and may play a role in autophagosome maturation (Cao and Klionsky, 2007).

Rab5, known for its role in conferring membrane identity to early endosomes and for recruiting effector proteins required for their maturation into Rab7-positive late endosomes, has also been shown to interact with and activate Vps34 leading to generation of phosphatidylinositol 3-phosphate (PI3P) (Christoforidis et al., 1999; Shin et al., 2005). This function is required for the early stages of autophagosome formation (Ravikumar et al., 2008). The class III PI3-K complex produces PI3P, which is required for recruitment of effector proteins and leads to phagophore membrane elongation and autophagosome maturation by enhancing the ubiquitin-like conjugation of Atg12 to Atg5. A second ubiquitin-like conjugation pathway involving microtubule protein 1 light chain 3 (MAP1-LC3/LC3/Atg8) results in the modification of LC3-I to LC3-II and its recruitment from the cytoplasm to autophagosomes. LC3 can mediate membrane tethering and hemifusion, which may be required for elongation and maturation of a phagophore into an autophagosome (Nakatogawa et al., 2007).

### Maturation and fusion

Autophagosomes can form anywhere in the cytoplasm and can then move bidirectionally along microtubules with a bias towards the microtubule organising centre, where they are more likely to encounter lysosomes (Jahreiss et al., 2008; Kimura et al., 2008). Autophagosome maturation and fusion is dependent on microtubules and dynein motor protein function (Kochl et al., 2006; Ravikumar et al., 2005; Webb et al., 2004). Autophagosomes fuse with endosomes and lysosomes which deliver more components, including lysosomal hydrolases, which are critical for protein and lipid degradation. These fusion reactions are dependent on the function of a number of proteins also known to be important in endosome–lysosome fusion reactions. ESCRT (endosomal sorting complex required for transport) proteins are required for correct fusion between autophagosomes and endosomes/lysosomes, although the mechanisms are unclear. It may be that they are required for recruitment of fusion machinery, such as Rab7 and SNAREs to endosome membranes (Filimonenko et al., 2007; Lee et al., 2007; Rusten et al., 2007; Urwin et al., 2010). SNAREs are involved in membrane tethering and fusion events and certain SNARES are important for autophagosome–lysosome fusion (Atlashkin et al., 2003; Furuta et al., 2010). Rab7 is a small GTPase that recruits a number of effectors that are required for fusion (Gutierrez et al., 2004; Jager et al., 2004), since loss of function of many Rab7 effectors leads to an accumulation of autophagosomes. Related to this, defects in lysosome acidification by genetic or drug treatments also prevent autophagosome clearance (Ramachandran et al., 2009).

## Autophagy malfunction and neurodegenerative diseases

Genetic studies using mice have highlighted the importance of constitutive autophagy in post-mitotic cells such as neurons (Hara et al., 2006; Komatsu et al., 2006), where it has a role removing aggregate-prone proteins that are toxic for the cell (Levine and Kroemer, 2009). Mice deficient for neuronal Atg5 or Atg7 (both key autophagic genes) develop progressive deficits in motor function that are accompanied by an accumulation of cytoplasmic inclusion bodies in neurons. It is important to stress that autophagy is clearing normal, soluble proteins in these situations. These results demonstrate that constitutive autophagy is important in neurons and that clearance of diffuse cytosolic proteins through basal autophagy prevents the accumulation of abnormal proteins, which may disrupt neural function (Hara et al., 2006; Komatsu et al., 2006). Here, we review how the impairment of different steps of the autophagic pathway may contribute to different neurodegenerative diseases.

### Initiation of autophagosome formation

Beclin-1 levels have been reported to decrease in an age-dependent way in human brains (Shibata et al., 2006), and this may lead to a decreased autophagic activity which could explain the effect of aging in neurodegeneration caused by aggregate accumulation. Affected brain regions of patients with Alzheimer's disease (AD) show reduced levels of Beclin-1 (Pickford et al., 2008). Heterozygous deletion of Beclin-1 in a mouse model of AD increased β-amyloid accumulation and neurodegeneration (Pickford et al., 2008).

Another key regulator of autophagy is the mammalian target of rapamycin (mTOR) kinase, which suppresses autophagy when it is activated (Levine and Klionsky, 2004). An abnormal activation of mTOR has been recently described in a mouse model of Lafora disease (LD) and in human tissue from patients suffering this neurodegenerative disorder (Aguado et al., 2010). LD is a progressive myoclonus epilepsy, characterized by the accumulation of polyglucosan inclusion bodies called Lafora bodies (Lafora and Glueck, 1911; Yokoi et al., 1975). The major genetic defect causing LD is the loss of function of a protein phosphatase called laforin (Minassian et al., 1998). Lack of laforin inhibits autophagy, associated with decreased autophagosome formation, decreased clearance of long-lived proteins, and accumulation of ubiquitinated proteins (Aguado et al., 2010).

Recently, we linked autophagy compromise to Parkinsons' disease (PD). PD is characterized by the accumulation of α-synuclein in aggregates. α-Synuclein accumulation is sufficient to cause PD, as this disease is seen in people with extra copies of the wild-type α-synuclein gene (Ross et al., 2008). We found that α-synuclein inhibits autophagosome formation in cell culture and *in vivo* (Winslow et al., 2010). This can account for many of the diverse cellular phenotypes seen in this disease, including intracytoplasmic protein accumulation, mitochondrial dysfunction and increased apoptosis. Since sporadic PD is also associated with α-synuclein accumulation, our data may have much wider implications.

### Cargo recognition

Autophagy has long been considered a non-selective bulk degradation pathway, but there is now evidence suggesting the existence of selective autophagy, which leads to degradation of specific organelles, proteins and pathogens (Kirkin et al., 2009; Kraft et al., 2009). p62, an ubiquitin-LC3-binding protein, is one of the molecules that links the cargo, protein aggregates or organelles, to the vesicle-forming machinery (Kim et al., 2008; Pankiv et al., 2007). Recently, a defect in cargo recognition mediated by p62 has been described in different cell models of Huntington's disease (HD) (Martinez-Vicente et al., 2010). HD is caused by an abnormally expanded polyglutamine tract close to the N-terminal end of the huntingtin protein. The mutated protein accumulates in aggregates within the cell, causing cell death (Imarisio et al., 2008). The mutant huntingtin can interact with p62, impairing its ability to recognize cargo aggregates and organelles. For this reason, though the synthesis of autophagosomes and their fusion to lysosomes is normal, their cargo content is decreased. This leads to an accumulation of aggregates and organelles like lipid droplets and mitochondria, which may contribute to neurodegeneration (Martinez-Vicente et al., 2010).

### Autophagosome–lysosome fusion

Dysfunction of ESCRT-III, either by depletion of its essential subunit mSnf7-2, or by expression of a mutant CHMP2B protein (another component of the complex) is associated with frontotemporal dementia linked to chromosome 3 (FTD3) and amyotrophic lateral sclerosis (ALS), both characterized by progressive neuronal accumulation of ubiquitin-positive protein aggregates (Parkinson et al., 2006; Skibinski et al., 2005). Studies in mature cortical neurons showed that depletion of ESCRT-III causes autophagosome accumulation and the inhibition of autophagic clearance of cytosolic proteins, protein aggregates and organelles (Filimonenko et al., 2007; Lee et al., 2007; Rusten et al., 2008). Similarly, depletion of ESCRT-I and ESCRT-II causes an accumulation of autophagosomes, suggesting that the normal ESCRT function is required for autophagosome–lysosome fusion (Lee et al., 2007).

### Lysosomal proteolysis

Once the autophagosomes fuse with the lysosomes, their cargo is degraded by the lysosomal hydrolases. Impairment of the activities of specific lysosomal hydrolases leads to the accumulation of the corresponding substrates inside lysosomes, a feature of most lysosomal storage disorders (LSDs) (Futerman and van Meer, 2004). A common cellular pathological feature in these diseases and their animal models is impaired autophagosome degradation, leading to the accumulation of autophagosomes and increased levels of LC3-II, and the accumulation of polyubiquitinated proteins and dysfunctional mitochondria, which are the putative mediators of cell death (Settembre et al., 2008). This have been described in different LSDs such as in Danon disease (Tanaka et al., 2000), GM1 gangliosidosis (Takamura et al., 2008), neuronal ceroid-lipofuscinoses (NCLs) (Koike et al., 2005), Pompe disease (Fukuda et al., 2006), multiple sulfatase deficiency (MSD) and mucopolysaccharidosis type IIIA (MPSIIIA) (Settembre et al., 2008).

Acidification of the new formed autolysosomes is needed for activation of the lysosomal hydrolases and effective proteolysis of substrates, and it is mediated by a vacuolar [H+] ATPase (v-ATPase) (Yamamoto et al., 1998). Recently, an impairment has been described in autolysosomal acidification in mouse models and cells from patients with early-onset familial Alzheimer's disease (FAD) due to mutations of preselinin-1 (PS1) (Lee et al., 2010), the most common cause of FAD (Cataldo et al., 2004). PS1 is needed for v-ATPase targeting to lysosomes, lysosome acidification, and proteolysis during autophagy (Lee et al., 2010).

### Consequences of autophagy compromise

There may be various consequences of impaired autophagy that are relevant for neurodegenerative diseases. For instance, a failure to clear dysfunctional mitochondrial may lower apoptotic thresholds for susceptible neurons. In addition, autophagy compromise leads to the accumulation of the ubiquitin-binding protein p62. Excessive levels of p62 impair the trafficking of ubiquitinated proteins to the proteasome, thereby leading to a secondary deficiency of the ubiquitin proteasome system in cells with autophagy compromise (Korolchuk et al., 2009). This leads to the accumulation of short-lived critical cellular regulators that are proteasome substrates, like p53, which cause stress and ultimately apoptosis when their levels are elevated. Thus, a secondary compromise of the flux of proteasome substrates may be an important factor regulating toxicity when autophagic activity is decreased. This study also helps explain why the aggregates formed when autophagy is compromised are decorated by antibodies to both ubiquitin and p62. Our data suggest that the p62 forms the seeds for these aggregates, which, in turn, sequester ubiquitin and proteins that are ubiquitinated, via the ubiquitin-binding domain of p62 (Korolchuk et al., 2009).

## Candidates for pharmacological induction of autophagy

### mTOR-dependent pathway

Chemical induction of autophagy protects cells against the toxic insults of aggregate-prone proteins associated with neurodegeneration by promoting their clearance. The first known drug identified as an autophagy inducer is rapamycin, which was already in clinical use for other indications. In mammalian cells, rapamycin inhibits the kinase activity of mTOR by forming a complex with the immunophilin FK506-binding protein of 12 kDa (FKBP12) that binds to and inactivates mTOR, leading to the upregulation of autophagy (Berger et al., 2006). Our studies have established that rapamycin treatment enhances the clearance of mutant huntingtin fragments, reduces aggregate formation and protects against toxicity in cell, *Drosophila* and mouse models of Huntington's disease (HD) (Berger et al., 2006; Ravikumar et al., 2006; Sarkar et al., 2009; Webb et al., 2003). We have also shown that rapamycin promotes the clearance of other intracytoplasmic, disease-associated, aggregate-prone proteins, including mutant proteins associated with spinocerebellar ataxias, mutant forms of α-synuclein implicated in PD, and mutant tau responsible for FTD, thereby attenuating their toxicity (Berger et al., 2006; Menzies et al., 2006; Williams et al., 2006). It is likely that autophagy regulates clearance of SDS-soluble species of these proteins, and that the formation of large aggregates visible by light microscopy is influenced by autophagy clearing the smaller aggregate precursors (Rubinsztein, 2006). In *Drosophila* models of these diseases, the benefits of rapamycin appear to be autophagy dependent, as this drug had no effects on the proteinopathy toxicity in flies expressing these mutant proteins when the activity of different autophagy genes was reduced (Berger et al., 2006; Pandey et al., 2007; Wang et al., 2009). These findings support the view that the primary benefits of this drug are mediated by autophagy and not by alternative mechanisms like disruption of the translational machinery. Consistent with the *Drosophila* data, the rapamycin analogue CCI-779 reduces both mutant huntingtin and ataxin-3 levels, thereby attenuating toxicity in mouse models of HD and spinocerebellar ataxia type 3, respectively (Menzies et al., 2010; Ravikumar et al., 2004).

Rapamycin treatment and autophagy upregulation also protects cells against pro-apoptotic insults that are independent of aggregates, for example, with staurosporine treatment in cell culture and paraquat toxicity in *Drosophila* (Ravikumar et al., 2006). Thus, rapamycin treatment may be useful in certain disease conditions (including various neurodegenerative diseases) where a slow but increased rate of apoptosis is evident, even if they are not associated with overt aggregate formation.

Recently, the delivery of the Beclin-1 gene was shown to induce autophagy and reduce amyloid and α-synuclein pathology in mouse models of AD and Parkinson/Lewy Body diseases, respectively (Pickford et al., 2008; Spencer et al., 2009). This approach, which is likely to be downstream or independent of mTOR, provides proof of principle for autophagy induction as a protective strategy in a wide range of neurodegenerative diseases.

### mTOR-independent autophagy activators

TOR proteins control several cellular processes besides autophagy, including repression of ribosome biogenesis and protein translation (Berger et al., 2006; Yang et al., 2005). These processes likely contribute to the complications seen with its long-term use.

Accordingly, we and others have embarked on a series of studies to identify novel autophagy-upregulating compounds and have discovered pathways that are independent of the target of rapamycin. Lithium, a mood stabilising drug used for the long-term treatment of affective disorders, facilitates the clearance of mutant huntingtin in HD cell and *Drosophila* models by reducing IP3 levels, and reduces mutant protein-associated aggregation and toxicity (Sarkar et al., 2005). Consistent with the role of IP3 in autophagy, pharmacological inhibition of the IP3R by xestospongin B also induces autophagy (Criollo et al., 2007). Additionally, sodium valproate and carbamazepine, which inhibit inositol synthesis and therefore decrease IP3 levels, also reduced the accumulation and aggregation of mutant huntingtin and its toxicity in HD cell models, and protected against neurodegeneration in *Drosophila* models of HD (Sarkar and Rubinsztein, 2008; Williams et al., 2008).

### A rational mechanism for combination treatment of mTOR-independent and mTOR-dependent pathways

Our laboratory investigated the prospect of a combination treatment strategy that would enable greater upregulation of autophagy, for example, by simultaneously inducing autophagy using mTOR-dependent and -independent pathways. By using dual treatment with rapamycin and lithium, we demonstrated that autophagy can be enhanced to a greater extent by the inhibition of both pathways, compared to saturating doses of drugs that inhibit each pathway individually (Sarkar et al., 2008). This strategy may allow a larger safety window before toxic effects from non-autophagy-related effects of each drug are seen. Proof of principle of this combination treatment approach *in vivo* has also been shown in HD fly models (Sarkar et al., 2008).

The rationale for enhancing autophagy using modulators of mTOR-dependent and mTOR-independent pathways may also be seen by the use of saturating doses of rapamycin with other mTOR-independent autophagy inducers.

### Screening of compounds—SMERs and various drugs

To explore other novel therapeutic agents capable of modulating autophagy for the purpose of treating neurodegeneration, a high-throughput small molecule screen was performed in yeast (Sarkar et al., 2007). From the 50,729 compounds screened, several small molecule inhibitors and enhancers (SMERs) of the cytostatic effects of rapamycin were identified. Among these, SMERs 10, 18 and 28 increased clearance of autophagy substrates, such as A53T α-synuclein and mutant huntingtin, and reduced huntingtin toxicity in the *Drosophila* HD model (Sarkar et al., 2007). This effect was independent of mTOR.

A further screen was then performed on a library comprising 253 FDA-approved drugs and pharmacologically active compounds by analyzing their effects on the clearance of known autophagy substrates (Williams et al., 2008). The screen revealed various autophagy enhancers such as L-type Ca2+ channel antagonists (e.g., verapamil, loperamide and amiodarone), the K+ATP channel opener minoxidil, and the Gi-signalling activators clonidine and rilmenidine, whose mechanisms of action were found to be linked in a potential cyclical fashion(Williams et al., 2008). These compounds induce autophagy by acting on the cAMP–Epac–PLCε–IP3-calcium pathway, described above. Overlapping drugs were also identified in an independent screen (Zhang et al., 2007). The drugs decrease mutant huntingtin aggregate levels via mTOR-independent autophagy and confer protection against toxicity in HD cell, fly and zebrafish models (Williams et al., 2008). A follow-up study has shown that rilmenidine enhances the removal of mutant huntingtin in a transgenic mouse model and also alleviates toxicity (Rose et al., 2010). This is exciting, since rilmenidine is a safe, centrally acting antihypertensive drug that acts on the same receptors to reduce blood pressure as it does to induce autophagy. These Gi-coupled imidazoline receptors are widely expressed in the brain. Thus, we believe this drug is a strong candidate to take forward into clinical trials in HD patients, given its safety and suitability for long-term use.

## Antioxidant drugs and autophagy

The appreciation of the relationships between autophagy and neurodegeneration has increased dramatically in the last decade. Many neurodegenerative diseases, like HD, exhibit increased oxidative stress, and antioxidants have been proposed as a rational therapeutic strategy. Indeed, there are at least 2 major trials of antioxidants in progress for HD. However, many antioxidant drugs inhibit autophagy in cells, primary neurons and in mice. By blocking autophagy, antioxidant drugs can increase the levels of aggregate-prone proteins associated with neurodegenerative disease, like mutant huntingtin. In fly models of Huntington's disease, the rescue seen with rapamycin is abrogated by antioxidant treatment and both antioxidant drugs and overexpression of superoxide dismutase can exacerbate the disease phenotype. Furthermore, antioxidants enhanced mutant huntingtin accumulation in our zebrafish model of HD (Underwood et al., 2010). Thus, the potential benefits of ROS scavengers in neurodegenerative diseases, for instance, as buffers against the toxic effects of increased reactive oxygen species, may be compromised by their autophagy-blocking properties. These studies also suggest that autophagy inhibitors may be broadly deleterious in such neurodegenerative diseases.

## Concluding remarks

In conclusion, while our understanding of autophagy continues to inform about pathogenic mechanisms and provide rational therapeutic strategies for neurodegenerative conditions, further studies on autophagy may also inform about treatment strategies and physiological processes, which were previously thought to be unrelated to protein clearance pathways. Clearly, the strategies may need to be tailored to fit the specific autophagy defects in particular diseases. For instance, diseases associated with autophagy impairment due to increased mTOR activity may be amenable to therapy with mTOR inhibitors. Additionally, care may be needed before upregulating autophagy when there is a blockade in autophagosome–lysosome fusion.
